# Epigenetics, Stress, and Their Potential Impact on Brain Network Function: A Focus on the Schizophrenia Diatheses

**DOI:** 10.3389/fpsyt.2014.00071

**Published:** 2014-06-23

**Authors:** Vaibhav A. Diwadkar, Angela Bustamante, Harinder Rai, Monica Uddin

**Affiliations:** ^1^Department of Psychiatry and Behavioral Neurosciences, Wayne State University School of Medicine, Detroit, MI, USA; ^2^Center for Molecular Medicine and Genetics, Wayne State University School of Medicine, Detroit, MI, USA

**Keywords:** schizophrenia, adolescence, risk, epigenetics, brain networks

## Abstract

The recent sociodevelopmental cognitive model of schizophrenia/psychosis is a highly influential and compelling compendium of research findings. Here, we present logical extensions to this model incorporating ideas drawn from epigenetic mediation of psychiatric disease, and the plausible effects of epigenetics on the emergence of brain network function and dysfunction in adolescence. We discuss how gene–environment interactions, effected by epigenetic mechanisms, might in particular mediate the stress response (itself heavily implicated in the emergence of schizophrenia). Next, we discuss the plausible relevance of this framework for adolescent genetic risk populations, a risk group characterized by vexing and difficult-to-explain heterogeneity. We then discuss how exploring relationships between epigenetics and brain network dysfunction (a strongly validated finding in risk populations) can enhance understanding of the relationship between stress, epigenetics, and functional neurobiology, and the relevance of this relationship for the eventual emergence of schizophrenia/psychosis. We suggest that these considerations can expand the impact of models such as the sociodevelopmental cognitive model, increasing their explanatory reach. Ultimately, integration of these lines of research may enhance efforts of early identification, intervention, and treatment in adolescents at-risk for schizophrenia.

## Introduction

Schizophrenia remains the most profoundly debilitating of psychiatric conditions (1, 2). General theories have struggled to capture the complexity of the disorder: genetic polymorphisms (3), neurodevelopment (4), and altered neurotransmission [dopamine (DA) and glutamate] (5, 6) have all being proposed as mediating factors in its emergence. A recently proposed “sociodevelopmental cognitive model” (7) has made compelling additions to the discourse on schizophrenia, with a specific emphasis on psychosis. A factorial combination of genetic and neurodevelopmental effects sensitize the DA system in early life. The disordered sensitivity subsequently leads to a disordered stress response that is further amplified by misattributed salience and paranoia. This cascading and recursive series of events eventually leads to the entrenchment of psychosis (and schizophrenia), explaining the life-long nature of the illness. This model is uniquely important because it integrates environmental, genetic, developmental, and molecular mechanisms (all converging on dysregulated DA release), providing a synthesis for several multi-disciplinary research agendas. Here, we attempt an incremental contribution to this synthesis suggesting that an expansion of this model may help elucidate the following:

(a) How do gene–environment interactions, effected by epigenetic mechanisms, mediate the stress response? The role of epigenetic mechanisms may be crucial in understanding why certain individuals at genetic risk eventually convert to schizophrenia but others with similar genetic vulnerability do not.
(b) In this context, the vexing problem of specific genetic at-risk populations is considered. Specifically, adolescents with one or both of whose parents have a diagnosis of schizophrenia form a “perfect storm” of genetic and neurodevelopmental contributors to risk for schizophrenia. These individuals present with extensive pre-morbid cognitive deficits (8) and sub-threshold clinical symptoms (9), yet a majority of them do not appear to develop the disorder. Whereas unexplained neurodevelopmental variation and resilience may explain this (10), we suggest that epigenetic mediation, particularly of genes mediating the stress response in adolescence, may explain some of this uncharacterized variance.
(c) Finally, we note the vast evidence of functioning brain network disruptions in schizophrenia, and the fact that these disruptions are now being characterized in at-risk populations, including children of patients, and suggest that epigenetic effects may mediate the shaping of functioning brain networks in the adolescent risk state, resulting in a highly variable and (currently) unpredictable pattern of conversion to psychosis (hence explaining the difficulty in estimating incidence rates of schizophrenia in at-risk groups).

In short, the proposed addendum motivates the role of epigenetics in the schizophrenia diathesis, the (potentially crucial) role of epigenetics in setting gene-expression levels that mediate the stress response, and ultimate causal (though presently unproven) effect on developing brain networks that sub-serve many of the cognitive functions impaired in schizophrenia. We note at the outset, that the proposed extensions remain speculative, yet seek to account for the relative under-representation of epigenetic considerations in schizophrenia-related research to date. In fact, epigenetics may provide a more proximate mediator of neuronal and behavioral effects than changes in the DNA sequence, and in turn these neuronal alterations may predispose individuals to schizophrenia, a question that has received comprehensive coverage in a recent canonical review (11). Moreover, the proposed additions also provide a prospective research impetus for studying particular sub-groups such as children of schizophrenia patients, a group that provides a particularly unique intersection of genetic risk, altered neurodevelopment, and environmental contributions (12–14). Finally, the notion of stress reactivity impacting brain network function is a particular extension of the seminal concept of “allostatic load” (15, 16), morphologic degeneration as a response to repeated adaptive responses to stress.

## Genetics, Development, Environment: An Array of Interactions

Schizophrenia is an “epigenetic puzzle” (17). Apart from the rare variant of the illness that is childhood onset schizophrenia (18), the typical manifestations of schizophrenia occur in late adolescence and early adulthood (1). This relatively late onset suggests that a seemingly intractable array of interactions between genetically endowed vulnerability, and environmental effects may amplify genetic predisposition, leading to post-natal effects on brain plasticity and development in the critical adolescent period (2, 19). The role of genes in mediating the emergence of the disorder is likely to be extremely complex. After all, genes do not code for complex psychiatric disorders but for biological processes (20). Thus, dysfunctional genetic expression is likely to lead to dysfunctional biological processes, with psychiatric disorders an emergent phenomenon in this causal pathway (20, 21). Moreover, the lack of complete concordance even in monozygotic twins (22, 23), suggests that genes primarily confer *vulnerability* to the illness and that other factors that mediate gene-expression during pre- and post-natal developmental, life span, and environmental effects play a significant role in the transition to the illness.

Several proximate environmental factors may be highly relevant as noted in the sociodevelopmental cognitive model. Stress – narrowly defined as a real or employed threat to homeostasis (24) – assumes particular importance, primarily because adolescence is a period of dynamic stress both in terms of substantive neurodevelopmental turnover (25), and environmental influence (26). Repeated stress exposure in particular during critical developmental periods exerts untenable biophysical costs. These costs typically referred to as allostatic load, increase vulnerability for somatic disease (27), and notably exert tangible biological effects. For example, glucocorticoid elevations that result from chronic stress have been associated with medial temporal lobe atrophy across multiple disorders including mood disorders, post-traumatic stress disorder, and schizophrenia (28–30). Beyond medial temporal lobe regional atrophy, the documented molecular effects in the prefrontal cortex are suspected to ultimately impact frontal–striatal brain networks (31, 32). Elevated DA release during acute stress (33) adversely affects prefrontal pyramidal cells leading to a series of degenerative molecular events. The resultant dendritic spine loss in the infra-granular prefrontal cortex results in reductions in prefrontal-based network connectivity, particularly on prefrontal efferent pathways (34). These molecular effects are likely to have mesoscopic expressions; among them disordered prefrontal cortex related brain network function and organization that are hallmarks of schizophrenia (3, 35–37).

## Stress and the Risk State for Schizophrenia

The risk state for schizophrenia offers a powerful framework for synthesizing multiple theoretical constructs of the disease (38), and disordered stress reactivity may play a key role in amplifying disposition for psychosis in the risk state (39). A critical challenge for high-risk research is navigating the relationship between multiple (and potentially non- or partially overlapping) risk groups each with different etiologies and defined based on different criteria (40). Here we consider prodromal subjects (41–46) in whom the role of stress has been heavily assessed, separately from adolescents with a genetic history of schizophrenia (including twins discordant for the illness and offspring of patients). The role of stress in the latter groups is relatively understudied. We note that the distinction does not imply exclusivity but rather criteria used to identify risk. Prodromal or clinical high-risk subjects (also on occasion referred to as “ultra high-risk”) are classified as such because they show non-specific yet considerably advanced clinical symptoms (47). Rates of conversion to psychosis within a short period after the emergence of clinical symptoms are high (estimates at 35%) (48). Genetic high-risk groups are identified typically on account of a family history of the illness itself; that is, not using clinical criteria. However, genetic high-risk groups may present with prodromal symptoms, hence these groups are not exclusive.

We will ultimately seek to drive our ideas in the direction of genetic risk in adolescence, largely because the prodromal question is heavily addressed in the sociodevelopmental model, whereas adolescent genetic risk is not. The adolescent genetic risk state presents a particularly vexing challenge, with substantial heterogeneity, and relative low rates of conversion to psychosis (9). The early identification of individuals who are likely to convert from the genetic risk state to actual schizophrenia (or psychosis?) thus remains a key issue to be addressed by future research efforts, as we propose here.

Prodromal subjects (sometimes referred to as “clinical high-risk”) present with a variety of symptoms that do not specifically warrant a diagnosis of schizophrenia, but include paranoia and impairment in social function. In general, prodromal patients have high rates of conversion to schizophrenia itself (48). For instance, multiple studies suggest that the average 12-month conversion rate in ultra high-risk samples not receiving any special anti-psychotic treatment is between 35 and 38% (48, 49). That a significant percentage of these individuals convert to psychosis is unsurprising because as noted, the prodromal state consists of highly advanced stage of clinical symptoms. Thus, these relatively non-specific symptoms that lead, and predict the presentation of the illness itself (38, 48, 50, 51) are considered the best clinical predictor of schizophrenia itself. Impaired neurobiology of the prodromal state is also relatively well understood: subjects are characterized by profound deficits in brain structure that are typically intermediate between healthy controls, and those observed in patients. Recent fMRI studies indicate substantive deficits in regional and brain network interactions (52–54) including frontal–striatal and frontal–limbic; cognitive and social neuroscience has established a crucial role for these networks in sub-serving basic mechanisms of memory, attention, and emotion. Heightened stress reactivity itself may be exacerbated by the presence of sub-threshold symptoms. For instance, prodromal subjects indicate heightened sensitivity to inter-personal interaction, an indirect measure of heightened stress (55), and a significant percent of prodromal subjects who have experienced trauma in their lives convert to psychosis (41). As noted, DA synthesis is increased in prodromal subjects, and the degree of synthesis is positively associated with the severity of sub-threshold clinical symptoms (56). Moreover, impaired stress sensitivity is also associated with a wide range of prodromal symptoms (44). The role of stress sensitivity, the hypothalamic–pituitary–adrenal (HPA) axis, and its impact on brain structures, has been heavily treated in the empirical and theoretical literature (43, 45, 57–59).

In contrast to the prodromal state, which includes individuals with a degree of existing symptoms, the genetic high-risk state encompasses individuals who are defined by having one (or more) parent(s) with schizophrenia, and who themselves may or may not evince symptoms of the disorder. The genetic high-risk state constitutes a partial complement of the clinical high-risk or prodromal state (these samples are often “enriched” by subjects with a family history of schizophrenia or psychosis providing overlap) (60). Genetic distance from a schizophrenia patient is a strong predictor of risk for the disease, and of the degree of biological impairments including brain structure, function, and behavior (61, 62). For example, children of schizophrenia patients being reared by the ill parent constitute a very particular and enigmatic high-risk sub-group (9, 13). These individuals have a genetic loading for the disease, but are also likely exposed to increased environmental stressors by virtue of being raised by their ill parent. Unlike with prodromal patients, conversion to psychosis in genetic high-risk groups is variable and lower.

Three principle longitudinal genetic high-risk studies are informative regarding lifetime incidence of schizophrenia in these groups. Between them, the New York (63), the Copenhagen high-risk projects (64), and a notable Israeli study (65) have provided evidence of lifetime incidences of narrowly defined schizophrenia at between 8 and 21%. While low, these rates constitute significantly elevated incidence rates relative to the sporadic incidence in the population (~1–2%). However, these rates are still notably lower than conversion rates in prodromal populations, a discrepancy that is somewhat surprising because the developmental psychopathology that characterizes prodromal patients is the very same one that is in play in adolescent high-risk subjects (45, 46). Subjects at genetic risk also show increased HPA axis sensitivity (59, 66), similar to what is observed in prodromal subjects, though the relationship to regional measures of brain integrity (e.g., pituitary size), is highly variable, and perhaps not informative as a biomarker (67). Heterogeneity is a cardinal characteristic of genetic risk groups (68, 69). Significant percentages of these subjects show attention deficits, working memory impairment, emotion dysregulation, and sub-threshold symptoms including negative symptoms (9, 70–75). Notably each of these cognitive, emotional, and clinical domains is highly impacted by stress sensitivity in adolescence (76, 77). Adolescent risk subjects also present with increased frequency of sub-threshold clinical symptoms including schizotypy and both positive and negative symptoms such as anhedonia (78–80), some of which have been associated with perceived stress (81, 82).

Understanding of altered DA synthesis in genetic risk groups is limited. A recent study in twins discordant for schizophrenia showed no increase in the elevation of striatal DA synthesis in the healthy twin (83) though the age range was well past the typical age of onset of the illness, and the healthy twin must retrospectively be classified as “low risk.” It is plausible the elevated striatal DA is not a marker of genetic risk *per se*, but might distinguish between adolescent sub-groups. Given that animal models and human studies have been highly informative in elucidating the impact of stress on neurobiology (32, 84), it is plausible that these effects might be quantifiable in neuroimaging data derived from such models in the context of risk for schizophrenia.

## Brain Network Dysfunction in the Adolescent Risk State for Schizophrenia

The origins of psychiatric disorders lie in adolescence (85, 86), a developmental stage characterized by a unique set of vulnerabilities, where highly dynamic neurodevelopmental processes intersect with increasing environmental stressors (26, 87). The idea of “three-hits” in schizophrenia, which includes pre-natal insults (e.g., obstetric complications, exposure to infections *in utero*), neurodevelopmental processes and disease-related degeneration, predicts the emergence of reliable and identifiable abnormalities through the life span (10, 88, 89). Notably, the period from birth to early adulthood is characterized by significant potential for epigenetic dysfunction that can increase symptom severity, beginning with the emergence of sub-threshold symptoms in adolescence, and culminating (in some individuals) in psychotic symptoms in young adulthood (11). Moreover, brain network development remains highly tumultuous in this period and disordered brain network dynamics are likely to be a cardinal biological characteristic in adolescents at genetic risk for the illness (13).

Disordered frontal–striatal and frontal–limbic brain network interactions, a defining characteristic of schizophrenia (90, 91), are increasingly established in the adolescent genetic risk state. These interactions are well-understood for working memory and sustained attention, both domains particularly associated with these regions (92), with risk for schizophrenia (70), and with DA (93, 94). During working memory, adolescents at genetic risk for schizophrenia show inefficient regional responses as well as network interactions in frontal and striatal regions. During working memory-related recall, at-risk subjects hyper-activate frontal–striatal regions, specifically for correctly recalled items (95), an effect highly consistent with what has been documented in schizophrenia itself (96, 97) and with large studies assessing the relationship between genetic risk and prefrontal efficiency (98).

More impressively, network interactions are also inefficient. For instance, the degree of modulation by the dorsal anterior cingulate, the brain’s principle “cognitive control” structure (99), during working memory is significantly increased in at-risk subjects (100). Thus, when performing the task at levels comparable to typical control subjects, control-related “afferent signaling” from the dorsal anterior cingulate cortex is aberrantly increased in adolescents at genetic risk. This evidence of inefficient pair-wise network interactions is highly revealing of “dysconnection” in the adolescent risk state. Similar results have been observed in the domain of sustained attention, where again, frontal–striatal interactions are impaired in the risk state (80, 101). Genetic high-risk subjects are also characterized by disordered “effective connectivity” estimated from fMRI signals. Effective connectivity is noted as the most parsimonious “circuit diagram” replicating the observed dynamic relationships between acquired biological signals (102). Recent evidence suggests reduced effective thalamocortical (54) and frontal–limbic (103) effective connectivity in genetic risk groups. These and other studies establish a pattern of general brain network dysfunction in adolescents at genetic risk for schizophrenia, suggesting that dysfunction in cortical networks is a plausible “end-point” in a cascade of genetic and neurodevelopmental events.

However, this story on brain networks is incomplete, because these high-risk groups present with considerable heterogeneity in sub-clinical symptoms, and recent evidence suggests that this heterogeneity predicts fMRI responses. For example, high-risk subjects with sub-threshold negative symptoms show attenuated responses to rewarding social stimuli, particularly in regions of the limbic system, including the amygdala and the ventral prefrontal cortex (75). This pattern of responses is in fact similar to those seen in patients with frank depression, and suggests additional compelling evidence in support of stress mediating the emergence of negative symptoms that in turn affect functioning brain networks (44, 104–107).

## Pathways and Epigenetic Mediation

Psychological stress is a major mediator of externally experienced (i.e., environmental) events, with relevance to both the central and peripheral nervous systems (108). Stress induces the release of corticotrophin releasing factor that activates the HPA axis to produce cortisol, and the sympathetic nervous system to produce norepinephrine and epinephrine. In some individuals, the initiation of an acute, adaptive “fight-or-flight” response in the face of threatening events becomes persistent and pathological. How this failure to return to homeostasis occurs in only a subset of individuals, resulting in a psychopathological state, remains to be fully elucidated. Stress is a clear risk factor for schizophrenia (109), and the biologic mechanisms linking stress, schizophrenia, and risk for schizophrenia are still being comprehensively characterized.

One candidate factor that may be a mediator in this causal chain is epigenetics, a field of increasing interest in mental illness, including risk for schizophrenia (110–112). Epigenetics, a term proposed nearly 70 years ago by Conrad Waddington, was born out of the terms “genetics” and “epigenesist,” narrowly referring to the study of causal relationships between genes and their phenotypic effects (113), but more recently associated with changes in gene activity independent of the DNA sequence, that may or may not be heritable, and that may also be modified through the life span. Epigenetic factors include DNA methylation which in vertebrates typically involves the addition of a methyl group to cytosine where cytosine and guanine occur on the same DNA strand; histone modifications, involving the addition (or removal) of chemical groups to the core proteins around which DNA is wound; and non-coding RNAs such as microRNAs (miRNAs), which bind to mRNAs to suppress gene-expression posttranscriptionally. Among these several mechanisms, DNA methylation is the most stable and the best studied within the context of psychiatric disorders, including schizophrenia, although emerging work suggests that miRNAs, which target multiple mRNA transcripts, serve as master regulators of developmental gene-expression patterns, and are responsive to stress (114), play an etiologic role in SCZ (115).

As mounting evidence fails to conclusively link individual genes to *specific* mental illnesses (116), epigenetic effects during critical developmental periods assumes increasing significance (11). In such a model, genetic etiology may be expressed in differentiated psychiatric phenotypes because epigenetic factors changing in response to external experiences vary across these phenotypes. Indeed, as potential regulators of DNA accessibility and activity, epigenetic factors through influences on gene-expression, offer a mechanism by which the environment – and, in particular, one’s response to the environment – can moderate the effects of genes (117). In the context of schizophrenia, models suggest that epigenetic deregulation of gene-expression at specific loci is highly unlikely, again given the highly polygenic nature of the illness. Rather, epigenetic effects may progressively impact gene-expression in salient neurodevelopmental gene networks during critical developmental periods, in response to environmental inputs (11). For example, the loss of synchronal activity of GABAergic interneurons in the prefrontal cortex might result from environmental stressors such as cannabis (118), which interact with the expression of vulnerability genes such as GAD1 that control GABA synthesis (119).

Previous work has shown that glucocorticoids (GC) such as cortisol induce epigenetic, DNA methylation changes in HPA axis genes (e.g., FK506 binding protein 5, *FKBP5*), both in neuronal [i.e., hippocampal (120, 121)] and peripheral [i.e., blood (121–123)] tissues, as well as in additional cells relevant to the HPA axis [i.e., pituitary cells (120)]. Moreover, GC-induced DNA methylation changes persist long after cessation of GC exposure (121–123), suggesting that stress-induced GC cascades have long lasting consequences for HPA axis function that may be accompanied by behavioral (mal)adaptations (121, 124).

These epigenetic mechanisms are of relevance to the previously noted role of stress as a major contributor in the emergence of cognitive impairments in first episode psychosis, in particular resulting from high stress sensitivity in this group (125). Stress sensitivity, a tendency to experience negative affect in response to negative environmental events (126), is a well-established risk factor for psychopathology (127), including schizophrenia (44, 128). This role has been clarified in recent work using experience sampling methods (ESM), where participants in prospective studies note their life experiences in real time. Using a twin-study design in a large longitudinal cohort of mono- and dizygotic twins, participants recorded multiple mood and daily life events with stress sensitivity defined as an increase in recorded negative affect to event unpleasantness. Notably, stress sensitivity showed relatively little genetic mediation and was almost exclusively environmentally determined (126). Whereas non-ESM investigations and some animal studies in models of schizophrenia (129) suggest a genetic, heritable component, the majority of variance still appears to be environmentally determined (130, 131). Thus, stress sensitivity is a labile characteristic that can change in response to environmental experiences to alter risk for psychopathology. Tracking epigenetic changes in stress-sensitive genes of the HPA axis, as well as additional stress-sensitive genes that interact with the HPA axis, might enable identification of a biologic mechanism that mediates risk for, and the emergence, of schizophrenia. Indeed, strong signatures of gene-expression differences in stress-related genes have been recently identified in post-mortem brain tissue in a manner that distinguishes schizophrenia patients from controls and from individuals with other psychiatric disorders (132). Many of these are likely accompanied by DNA methylation differences, as has been reported by studies performed on related genes in animal models (133).

Emerging evidence suggests that brain endophenotypes, as well as psychiatric outcomes, can be predicted by peripheral DNA methylation measurements. Notably, genes belonging to the HPA axis, as well as DA- and serotonin (5HT)-related genes, whose products interact those of the HPA axis, shape the stress response (109, 134, 135) and are known to show psychopathology-associated differences in blood (136–138). For example, recent work has shown that leukocyte DNA methylation in the serotonin transporter locus (*SLC6A4*) was higher among adult males who had experienced high childhood-limited physical aggression; moreover *SLC6A4* DNA methylation was negatively correlated with serotonin synthesis in the orbitofrontal cortex, as measured by positron emission tomography (PET) (139). Similarly, leukocyte DNA methylation in the promoter region of the *MAOA* gene – whose product metabolizes monoamines such as serotonin and DA, is negatively associated with brain MAOA levels as measured by PET in healthy male adults (140). Structural imaging data analyses in relation to the *FKBP5* locus discussed above have identified a negative association between DNA methylation in peripheral blood and volume of the right (but not left) hippocampal head (121). This observation is particularly noteworthy, as it suggests that lower *FKBP5* DNA methylation in peripheral blood is associated not only with altered stress sensitivity (as indexed by a glucocorticoid receptor sensitivity assay within the same study), but also with structural brain differences in a brain region known to mediate stress reactivity (121). Finally, investigation of the *COMT* locus, a gene encoding an enzyme critical for degradation of DA and other catecholamines, has shown that, among Val/Val genotypes, subjects (all healthy adult males) with higher stress scores have reduced DNA methylation at a CpG site located in the promoter region of the gene (141). Moreover, DNA methylation at this site was positively correlated with working memory accuracy, with greater methylation predicting a greater percentage of correct responses (with results again limited to analysis of the Val/Val subjects); furthermore, fMRI demonstrated a negative correlation between DNA methylation at this site and bilateral PFC activity during the working memory task (141). Additional analyses showed an interaction between methylation and stress scores on bilateral prefrontal activity during working memory, indicating that greater stress, when combined with lower methylation, are associated with greater activity (141).

This last finding is especially noteworthy, because whereas stress–DNA methylation interactions have been reported for other stress-sensitive loci (142), the referenced study represents a direct demonstration of a heterogeneity in stress load that, when moderated by DNA methylation, impacts working memory. Clearly, greater stress and lower *COMT* DNA methylation correlate with reduced efficiency of prefrontal activity (141). This mechanism may be explained by the fact that disordered stress responses following prolonged stress exposure induces hyper-stimulation of prefrontal DA receptors (143, 144) that may be mediated by prefrontal glutamate neurotransmission (145). This hyper-stimulation in turn appears to affect the receptive field properties of prefrontal neurons during working memory (94). Patterns of network dysfunction in the genetic risk state may reflect brain network sensitivity to stress in the “pre-morbid” risk state that may be under as yet undiscovered epigenetic control. Thus, much of the unaccounted variance in schizophrenia previously construed as genetic, may likely be epigenetic (11, 146). Is it possible to assess epigenetic factors mediating the stress response in risk for schizophrenia, and the effects on brain network function?

**Figure 1:**
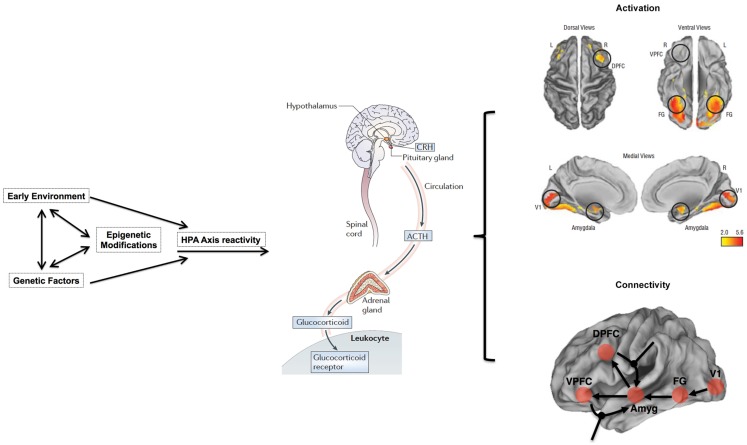
**Overview of working model**. HPA axis reactivity is determined both by intrinsic genetic factors and stressful environmental (including pre-natal) experiences. Stressful exposures induce a glucocorticoid (i.e., cortisol) cascade that then induces DNAm changes in HPA axis genes in the blood. These changes are expected to be more pronounced in at-risk adolescents, particularly those who may already exhibit sub-clinical psychopathology, such as negative symptoms. Risk-associated, blood-derived DNAm differences in HPA axis and related stress sensitivity genes are hypothesized to index metrics of brain function including activation patterns and effective connectivity in stress-sensitive brain regions. The activation patterns are reproduced from Diwadkar (13) and reflect engagement of an extended face-processing network in controls and high-risk subjects during a continuous emotion-processing task. These activations are most likely generated by complex dynamic interactions between brain networks that are represented in the figure below. The figure presents a putative combination of intrinsic connections between brain regions activated during such a task, and the contextual modulation of specific intrinsic connections by dynamic task elements. The role of effective connectivity analyses is to recover and estimate parameter values for intrinsic and modulatory connections that a) may be different in the diseased or risk state and b) may plausibly be under epigenetic mediation. The figure is adapted and reprinted from: Mehta and Binder (124), with permission from Elsevier; adapted by permission from Macmillan Publishers Ltd.: Frontiers in Neuropsychiatric Imaging and Stimulation (108). Reproduced with permission, Copyright © (2012) American Medical Association. All rights reserved.

The influence of stress on DNA methylation on HPA axis genes in blood is well established (121–123). Indeed, blood disperses GC hormones produced by the HPA axis throughout the body, which then regulates gene-expression in virtually all cell types (108). Thus, the broad reach of HPA axis activity, together with evidence that blood-derived DNA methylation in HPA axis genes is altered through stress (121, 147), provides ample biologic and clinical plausibility to our proposed hypothesis that stress sensitivity, measured in the periphery, can serve as an important – perhaps even predictive – index of transition from the genetic risk state into actual schizophrenia. Importantly, although GCs also influence DNA methylation and gene-expression in the CNS and neuronal cells (120, 121), our model does not suppose that this epigenetic measure in CNS tissues will match those in the periphery; rather, it proposes that DNA methylation in stress-sensitive, HPA-axis genes in the periphery will index the known dysregulation in brain function and connectivity in stress-sensitive regions of the brain among adolescents at genetic risk. Figure 1 provides an overview of an integrative approach and builds on previous considerations of epigenetic mechanisms in developmental psychopathology (11).

Existing data support the hypothesis that schizophrenia-associated DNA methylation differences exist in stress-sensitive genes. Table 1 summarizes results from existing genome-scale studies that have been conducted in blood and brain in relation to schizophrenia, focusing specifically on the HPA axis genes involved in the glucocorticoid receptor complex (148), as well as representative DA- and serotonin-related genes, and genes that produce DNA methylation and have been shown to be responsive to glucocorticoid induction in both the brain and periphery [i.e., DNA methyltransferase 1, *DNMT1*; (120)]. As can be seen from the table, all of the genes show SCZ-related DNA methylation differences in brain derived tissue (149), and the majority (four of five) of GC-receptor chaperone complex genes show DNA methylation differences in the blood as well. Although we have limited our analysis to genome-wide studies of DNA methylation, additional candidate gene studies have linked stress-sensitive mental disorders to methylation differences in blood (142, 150, 151), suggesting that similar findings may be forthcoming for schizophrenia as additional studies are completed. Importantly, among these genes, some (but not all) have shown that DNA methylation levels can vary depending on local [e.g., Ref. (141)] or distal [e.g., Ref. (121)] DNA sequence variation – so-called “methQTLs” (methylation quantitative trait loci). Thus, as evidence accumulates regarding the existence of methQTLs, we note that analyses based on these proposed genes should take these into consideration.

**Table 1:** **Summary of genome-wide studies reporting differential DNA methylation^a^ (DM) within stress-sensitive genes in blood or brain**.

Gene name	Pathway	Studies in blood					Studies in brain				
		Reference	Blood	Blood cell	Method	Data available?	Reference	Brain	Brain tissue	Method	Data available?
*COMT*	Dopamine catabolism	(152)*	Increased (cg13175282, cg06860277) DNA methylation in SCZ patients	Whole blood	450 K	GEO	(149)	Increased DNA methylation (cg12457376) in SCZ patients; DM between SCZ sub-groups, increased (cg00107488) and decreased (cg12728623, cg07579946, cg04856117, cg06787004) DNA methylation	Frontal cortex	450 K	Three supplemental tables including all DM CpGs
*DNMT1*	DNA methylation	NA	No SCZ-related DM reported in genome-wide blood-based studies to date	NA	NA	NA	(149)	Decreased DNA methylation (cg06128182, cg01347596) in SCZ patients; DM between SCZ sub-groups, increased (cg21892967) and decreased (cg12053136 and cg26705765) DNA methylation	Frontal cortex	450 K	Three supplemental tables including all DM CpGs
*FKBP4*	GC-receptor chaperone complex	(152)*	Decreased (cg15260466) DNA methylation in SCZ patients	Whole blood	450 K	GEO	(149)	Increased DNA methylation (cg00779206) in SCZ patients; increased (cg00779206) DNA methylation between SCZ sub-groups	Frontal cortex	450 K	Three supplemental tables including all DM CpGs
*FKBP5*	GC-receptor chaperone complex; HPA axis gene	(152)*	Decreased (cg25114611) DNA methylation in SCZ patients	Whole blood	450 K	GEO	(149)	DM between SCZ sub-groups. Increased (cg19226017, cg17030679, cg07061368) and decreased (cg14284211 and cg01294490) DNA methylation.	Frontal cortex	450 K	Three supplemental tables including all DM CpGs
*HSP90*	GC-receptor chaperone complex	(152)*	Increased (cg10833014 HSP90AA1) and decreased (cg07086455 *HSP90AA1*) DNA methylation in SCZ patients	Whole blood	450 K	GEO	(149)	Increased *HSP90AA1* (cg02017208) DNA methylation in SCZ patients	Frontal cortex	450 K	Three supplemental tables including all DM CpGs
		(153)	*HSP90AA1* hypomethylation (cg04281268) in First Episode SCZ patients	Peripheral blood cells	27K	Included supplement of the 603 DM CpGs					
*NR3C1*	GC-receptor; HPA axis gene	(152)*	Decreased (cg06968181 and cg17617527) DNA methylation in SCZ patients	Whole blood	450 K	GEO	(149)	Decreased (cg06613263 and cg07733851) DNA methylation between SCZ sub-groups	Frontal cortex	450 K	Three supplemental tables including all DM CpGs
*PTGES3*	GC-receptor chaperone complex	NA	No SCZ-related DM reported in genome-wide blood-based studies to date	NA	NA	NA	(149)	Decreased DNA methylation (cg20253639) in SCZ patients	Frontal cortex	450 K	Three supplemental tables including all DM CpGs
*SLC6A3*	Dopaminergic system	(154)*	Schizophrenia-associated DNA methylation (increased beta 0.05 avg) differences in discordant monozygotic twins	Whole blood	27 K	Only list top 100 DM CpGs	(149)	Decreased DNA methylation (cg01204634, cg05030481, cg24756227) in SCZ patients; decreased (cg24756227, cg16392193, cg16180821) DNA methylation between SCZ sub-groups	Frontal cortex	450 K	Three supplemental tables including all DM CpGs
		(153)	Hypomethylation (cg26205131) in first episode schizophrenia patients	Peripheral blood cells	27 K	Included supplement of the 603 DM CpGs					
		(152)*	Increased (cg1161677) and decreased (cg22659953) DNA methylation in SCZ patients	Whole blood	450 K	GEO					
*SLC6A4*	Serotonergic system	NA	No SCZ-related DM reported in genome-wide blood-based studies to date	NA	NA	NA	(149)	Decreased DNA methylation (cg03363743) in SCZ patients; decreased (cg26126367 and cg03363743) DNA methylation between SCZ sub-groups	Frontal cortex	450 K	Three supplemental tables including all DM CpGs

^a^DM based on adjusted *p*-values except where indicated (*).

*Gene-expression omnibus (GEO) is a public repository of functional genomic data accessible via NCBI*.

*The Horvath data were analyzed using GEO2R*.

## Conclusion

Incorporating epigenetic considerations into the sociodevelopmental model might provide a particular powerful explanatory framework for understanding genetic risk in adolescence. Regressive pressures from a combination of fixed genetic vulnerability for schizophrenia and epigenetic effects during adolescence are most likely to impact the development of neuronal network profiles (155, 156). As we noted earlier, advances in the analyses of fMRI signals now permit the estimate of effective connectivity and dysconnectivity between healthy, clinical, and at-risk populations, providing a significant framework for exploring brain dysfunction using *a priori* hypothesis (157). A focus on frontal–striatal and frontal–limbic dysconnectivity may be particularly warranted. A disordered stress response may cleave apart frontal–striatal and frontal–limbic neuronal network profiles in high-risk adolescents, providing a convergence of biological markers across multiple levels (genetic, epigenetic, and brain networks). Here, we have proposed that increased stress sensitivity (which can be indexed in the periphery) can help to unpack the heterogeneity among individuals at genetic high-risk of SCZ when linked to a strongly validated finding in genetic risk populations, namely brain network dysfunction. This framework may help to identify, among individuals at high genetic risk for SCZ, a subset who are likely to go on to develop the disorder. Our focus on stress-relevant genes does not exclude the possibility that genes in other pathways (e.g., dopaminergic, serotonergic, glutamatergic) may also be important; indeed, this focus may be considered a limitation of the proposed hypothesis. However, we believe that our proposed framework is a logical starting point for merging central and peripheral indicators of the potential for SCZ among HRS individuals. This framework may help extend the sociodevelopmental cognitive model into the realm of high-risk research. The presence of non-specific, sub-threshold symptoms continues to remain a significant clinical challenge for disorders such as schizophrenia and bipolar disorder (38, 158). Early intervention strategies will be boosted if biological markers can be interlinked to identify ultra high-risk adolescents. Our intent is to motivate this search for biological convergence hoping that this may lead to psychosis prediction and, ultimately, prevention.

## Conflict of Interest Statement

The authors declare that the research was conducted in the absence of any commercial or financial relationships that could be construed as a potential conflict of interest.
